# Tracing neuropathological signatures: *TARDBP* and *C9orf72* double mutations in a Sicilian family

**DOI:** 10.1093/hmg/ddaf147

**Published:** 2025-09-22

**Authors:** Pegah Masrori, Sandra O Tomé, Lieselot Dedeene, Gauthier Remiche, Hilde Van Esch, Dietmar Rudolf Thal, Philip Van Damme

**Affiliations:** Department of Neurology, Neuromuscular Reference Centre, University Hospitals Leuven, Herestraat 49, 3000 Leuven, Belgium; Department of Neurosciences, Experimental Neurology, and Leuven Brain Institute (LBI), KU Leuven, University of Leuven, Herestraat 49, 3000 Leuven, Belgium; Laboratory for Neuropathology, Department of Imaging and Pathology, Leuven Brain Institute (LBI) KU Leuven, Herestraat 49, 3000 Leuven, Belgium; Laboratory for Neuropathology, Department of Imaging and Pathology, Leuven Brain Institute (LBI) KU Leuven, Herestraat 49, 3000 Leuven, Belgium; Université libre de Bruxelles (ULB), Hôpital Universitaire de Bruxelles (H.U.B), CUB Hôpital Erasme, Service de Neurologie, Centre de Référence Neuromusculaire, Route de Lennik 808, 1070 Anderlecht (Brussels), Belgium; Center for Human Genetics, University Hospitals Leuven, Herestraat 49, 3000 Leuven, Belgium; Laboratory for Neuropathology, Department of Imaging and Pathology, Leuven Brain Institute (LBI) KU Leuven, Herestraat 49, 3000 Leuven, Belgium; Department of Pathology, University Hospitals Leuven, Herestraat 49, 3000 Leuven, Belgium; Department of Neurology, Neuromuscular Reference Centre, University Hospitals Leuven, Herestraat 49, 3000 Leuven, Belgium; Department of Neurosciences, Experimental Neurology, and Leuven Brain Institute (LBI), KU Leuven, University of Leuven, Herestraat 49, 3000 Leuven, Belgium

**Keywords:** ALS, C9ORF72, TAR DNA-binding protein 43, Neuropathology

## Abstract

Co-occurrence of double heterozygosity in *TARDBP* and *C9ORF72* is exceedingly rare in amyotrophic lateral sclerosis. While TARDBP mutations and *C9ORF72* hexanucleotide repeat expansions have each been independently implicated in disease pathogenesis, their combined effect on disease progression and neuropathology remains unclear. We present the first study documenting a patient harboring both a *TARDBP* mutation and a *C9ORF72* expansion, with comprehensive postmortem data available, to elucidate any additive or synergistic effects on disease course and pathological burden. Detailed clinical assessments tracked the patient’s progression, and neuropathological examination was performed postmortem. The presence and extent of TDP-43 pathology and other hallmark features were evaluated and compared to known patterns in carriers of isolated *C9ORF72* mutations. The patient’s clinical trajectory and pathological findings did not show evidence of a more aggressive disease course or heightened pathological burden attributable to the additional TARDBP mutation. Instead, the disease manifested in a manner consistent with other *C9ORF72* carriers, suggesting that double heterozygosity do not necessarily exacerbate ALS pathology.

## Introduction

The degeneration of motor neurons in amyotrophic lateral sclerosis (ALS) is characterized by intraneuronal TAR DNA-binding protein 43 (TDP-43) positive ubiquitinated inclusions (UBIs) in the majority of cases [1]. In up to 15% of patients, a causative gene mutation is found in one of the ALS-associated genes. Missense mutations in the *TARDBP* gene, which encodes the TDP-43, can cause ALS, but the most common cause is hexanucleotide repeat expansions in the chromosome 9 open reading frame 72 (*C9ORF72)* gene [2]. Many gene mutations underlying ALS display a reduced penetrance, and an oligogenic pattern with combinations of mutations in different genes is encountered in some patients. In such cases, the contribution of the individual mutations remains unclear and the impact on the neuropathology largely unexplored. Here, we report a non-consanguineous ALS pedigree with double mutations in *C9ORF72 TARDBP* and we describe the impact on the neuropathology.

**Figure 1:**
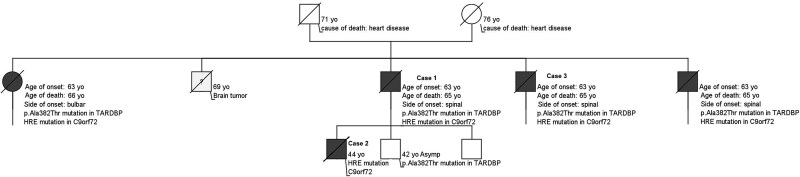
Pedigree.

Proband (Fig. 1 Pedigree, Case 1), a 63-year-old male presented with progressive lower limb weakness that first became noticeable approximately five months prior. He also reported balance difficulties but no substantial limitation in walking distance. However, for the past five months, he has required the support of a banister to aid in climbing stairs. Clinical examination revealed fasciculations in the calves and right deltoid. Muscle strength showed weakness in bilateral hip flexion and weakness in left hallux extension. The patient demonstrated slight bilateral weakness in walking on toes and heels. Areflexia was noted in the bicipital and radial reflexes on the right side, with no evidence of pyramidal signs. The patient showed no signs of cognitive or behavioral impairment. Routine blood tests indicated a mild chronic folic acid deficiency. Cervical and lumbar spine computed tomography revealed narrowing of the antero-posterior cervical canal. Nerve conduction studies and needle electromyography showed mild sensory neuropathy and active denervation signs in several lower limb muscles. Brain Magnetic resonance imaging (MRI) was unremarkable. Initial pulmonary function tests showed a forced vital capacity (FVC) of 3.45 L (91% of the predicted value), and treatment with riluzole was initiated. Prior to this, the proband’s brother (Fig. 1 Pedigree, Case 3), at the age of 65, had been diagnosed with spinal-onset ALS. The genetic testing, in which *C9ORF72*, *SOD1*, *TARDBP* and *FUS* are routinely tested, revealed that patient was heterozygous for the mutation c.1144G > A (p.Ala382Thr) in the *TARDBP* gene as well as *C9ORF72* HRE. Over approximately 18 months, his condition gradually deteriorated, leading to increasing difficulty with walking, climbing stairs, and respiratory failure. Ultimately, this progression necessitated the use of a walker, even within the confines of his home. The patient passed away at 65 with a moderate rate of advancement.

Proband’s son (Fig. 1 Pedigree, Case 2), a 44-year-old -male, presented with progressively worsening weakness in his right lower limb, accompanied by generalized fasciculations and cramps. Neurological examination revealed widespread fasciculations in all four limbs, with no apparent muscle atrophy. The patient exhibited paresis in the right lower limb, and hand-grip strength was diminished on the right side compared to the left. The jaw jerk reflex was brisk, but there were no other upper motor neuron signs. Sensory examination results were unremarkable. Routine blood tests indicated a mild chronic folic acid deficiency. Nerve conduction studies and needle electromyography demonstrated mild sensory neuropathy and active denervation signs in several lower limb muscles. Cervical and brain MRI were largely unremarkable, aside from slight cervical discopathies without cervical canal stenosis. Motor evoked potentials showed abnormal results with an increased threshold (75%) at the right first dorsal interosseous muscle and increased central conduction times in the lower limbs, with asymmetry favoring the right tibialis anterior. The diagnosis met the revised El Escorial criteria for ALS. Initial pulmonary function tests showed an FVC of 4.47 L (94% of the predicted value), and riluzole treatment was initiated. Genetic analysis revealed that the patient possessed an expanded hexanucleotide repeat in the *C9ORF72* gene. No mutation was detected in the *TARDBP* gene. Over the subsequent year, his condition deteriorated, requiring the use of a cane for ambulation. Approximately six months later, he became dependent on a wheelchair for mobility. At age 45, he developed rapidly progressive dysphagia and respiratory failure, leading to the initiation of non-invasive nocturnal ventilation. The patient became increasingly dependent on daily activities and died at the age of 46.

## Results

**Figure 2:**
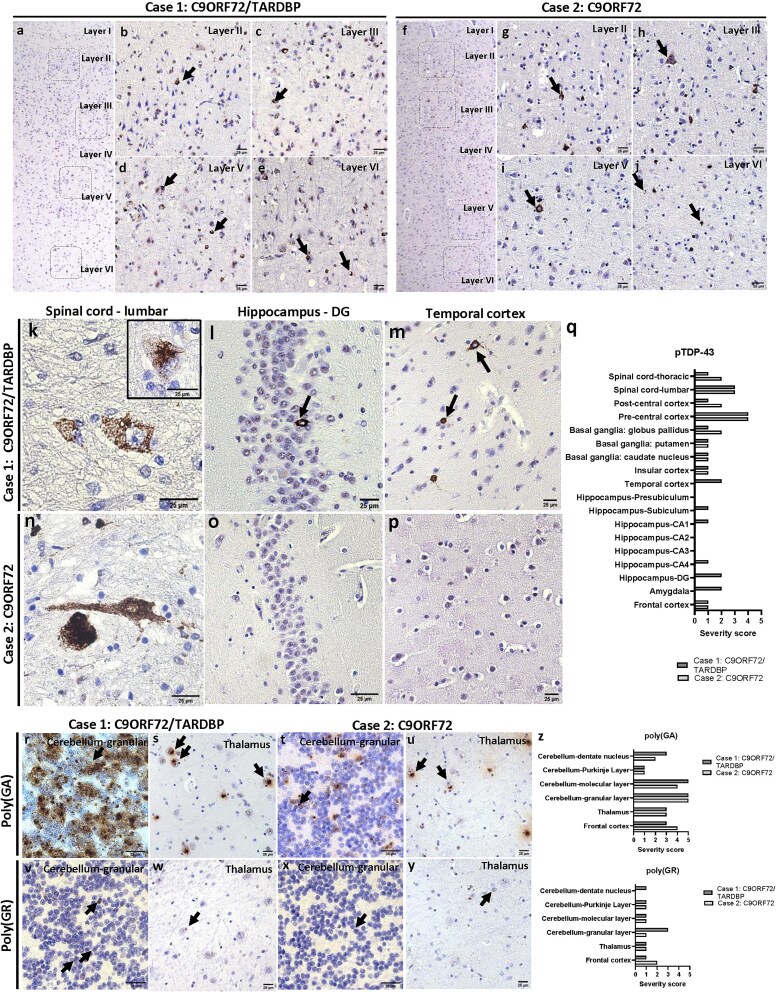
(a-j) Histological overview of the motor cortex stained with pTDP-43 (S409/S410) of (a-e) proband and (f-i) proband’s son. Insets at 200x of layers II, III, V and VI depict pTDP-43-positive neuronal and glial inclusions, and dystrophic neurites (arrows). (k-q) Immunohistochemical analysis of pTDP-43 (S409/S410) pathology of autopsy individuals in the lumbar spinal cord (k, n), granular layer of the dentate gyrus (DG) (l, o) and temporal cortex (m, p). Histological quantifications of pTDP-43 burden in all regions analyzed (q). (r-z) Immunohistochemical analysis of DPR pathology and respective quantifications. Poly(GA) pathological burdens (r-u) are similar between individuals (z). Proband displayed slightly higher burdens of poly(GR) compared to proband’s son, especially in the granular layer (r,v, z) and the dentate nucleus (z) of the cerebellum. Arrows denote inclusions. PMI = postmortem interval; NFT = neurofibrillary tangle.

**Figure 3:**
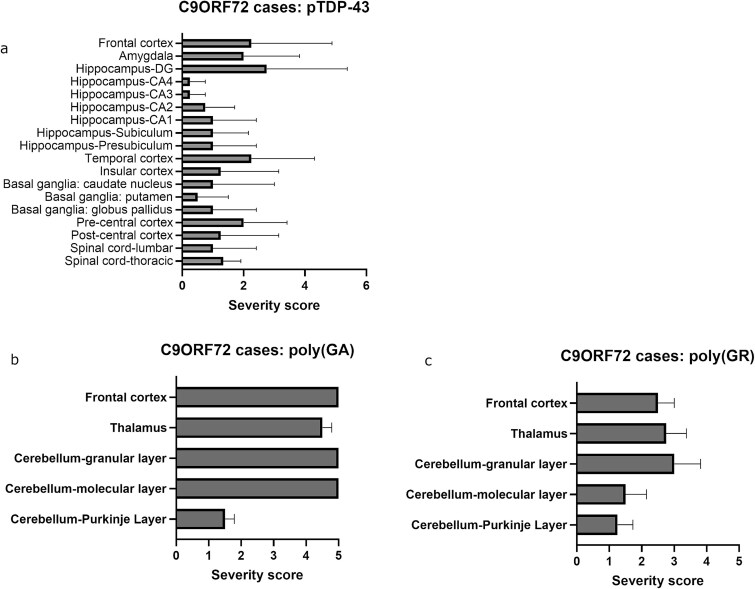
Immunohistochemical analysis of (a) pTDP-43 (S409/S410) and (b-c) DPR pathologies and respective quantifications of four other C9orf72 mutation carriers. Means with SEM are shown.

Brain and spinal cord autopsy followed by histological analysis were performed for proband (*C9ORF72/TARDBP* carrier) and proband’s son (*C9ORF72* carrier). The general neuropathological characteristics for both individuals are listed in Table 1. Macroscopically, both brains appeared normal. Moderate to severe MN loss was observed microscopically in the spinal cord of both patients. The diagnosis of ALS was confirmed for both individuals by the presence of pTDP-43 pathology in spinal cord MNs and neurons of the motor cortex (Fig. 2). Histopathological analysis revealed a pattern typically observed in *C9ORF72* and *TARDBP* mutation carriers [6]. Specifically, we observed extensive phosphorylated TDP-43 (pTDP-43) pathology in the six layers of the motor cortex (Fig. 2a-j), including neuronal cytoplasmic inclusions (Fig. 2b and g), diffuse neuronal pre-inclusions (Fig. 2h), glial inclusions (Fig. 2d and j) and dystrophic neurites (Fig. 2c and j). No striking morphological differences were observed between both individuals. In spinal cord tissue, pTDP-43-positive skein-like, dash-like and dense inclusions were observed in both individuals (Fig. 2k and n) [7, 8]. Of note, only proband exhibited pTDP-43 pathology in the amygdala, hippocampus, and temporal cortex (Fig. 2l and q), indicative of ALS stage 4, whereas proband’s son exhibited pTDP-43 pathology restricted to the spinal cord, pre and postcentral cortex, frontal cortex and basal ganglia, indicative of stage 3 [3]. Severity scores for pTDP-43 and dipeptide repeat proteins (DPRs) were evaluated as previously described [9]. Briefly, a semiquantitative grading system was used by counting the total amount of pathology (including neuronal cytoplasmic and intranuclear inclusions, dystrophic neurites, diffuse granular cytoplasmic staining and pan-nuclear DPR expression) in a 200x microscopic field considered as the ‘hotspot area’. DPR and TDP-43 pathology was rated as ‘0’ if no pathology was present, as ‘1’ if 1 to 3 pathological lesions were present in the hotspot area, as ‘2’ if 4 to 7 pathological lesions were present in the hotspot area, as ‘3’ if 8 to 20 pathological lesions were present in the hotspot area, as ‘4’ if 21 to 50 pathological lesions were present in the hotspot area and as ‘5’ if more than 50 pathological lesions were present. The severity scores for pTDP-43 pathology for both individuals analyzed per subregion are displayed in Fig. 2q. Four other *C9ORF72* expansion carriers were also analyzed for comparison (Table 2). Of note, these four individuals showed similar burdens of pTDP-43 pathology comparable to proband (double mutation carrier), suggesting that the presence of a *TARDBP* mutation did not influence the burden of pTDP-43 pathology (Fig. 3).

**Table 1:** Neuropathological characteristics of the human autopsy cases used in this study.

Case	Age at death	Sex	Disease duration (months)	PMI (h)	Brain weight	ALS pTDP-43 stage [3]	Aβ Phase [4]	Braak NFT stage [5]	Other neuropathologies
Proband *C9ORF72/TARDBP*	65	Male	24	3	1035 g	4	0	1	Atherosclerosis, small vessel disease
Proband’s son *C9ORF72*	46	Male	24	12	1477 g	3	0	1	AGD, ARTAG, small vessel disease

Abbreviations: AGD, argyrophilic grain disease; ARTAG, aging-related tau astrogliopathy.

**Table 2:** Neuropathological characteristics of the additional *C9ORF72* expansion carriers used in this study.

Case	Age at death	Sex	Disease duration (months)	PMI (h)	Brain weight	ALS pTDP-43 stage [3]	Aβ Phase [4]	Braak NFT stage [5]	Clinical Diagnosis	Other neuropathologies
#1	57	Male	19	20	1346 g	4	0	1	ALS	Atherosclerosis
#2	48	Male	18	24	1180 g	2	0	1	ALS	na
#3	55	Male	30	12	1200 g	4	0	3	ALS/FTD	Atherosclerosis
#4	58	Male	43	9	1280 g	4	0	1	FTD	Atherosclerosis, small vessel disease

Abbreviations: ALS = amyotrophic lateral sclerosis; FTD = frontotemporal dementia; na = not applicable.

DPR pathological burdens were evaluated in the frontal cortex, thalamus and different layers of the cerebellum, which are known to be vulnerable to DPR pathology in *C9ORF72* carriers [10, 11]. Both individuals had comparable poly(GA) pathological burdens in the different layers of the cerebellum, thalamus and frontal cortex (Fig. 2r-u and z), suggesting that the presence of a *TARDBP* mutation does not impact the severity of poly(GA) inclusions, probably because it occurs earliest in the disease progression. This is consistent with observations that poly(GA) is the most abundant DPR in *C9ORF72* carriers [10, 12]. Additionally, proband displayed denser and more abundant poly(GA) inclusions in the granular layer of the cerebellum (Fig. 2r), but this was not quantified, as both individuals still presented more than 50 lesions per 200x field, i.e., score 5. Both individuals showed generally sparse poly(GR) pathology, with proband showing higher burdens of poly(GR)-positive inclusions in the granular layer and the dentate nucleus of the cerebellum (Fig. 2v and z) compared to proband’s son (Fig. 2x and z). Of note, the remaining four *C9ORF72* carriers presented similar amounts of poly(GA) and poly(GR)-positive inclusions (Fig. 3b and c), on average even more severe than proband. Taken together, these data suggest that the presence of an additional mutation on the *TARDBP* gene does not impact the neuropathological burden of pTDP-43 or DPR pathology.

## Discussion

This study highlights the clinical and genetic complexity observed in ALS cases with co-occurring *C9ORF72* and *TARDBP* mutations. The phenotypic variability within this family may be partly attributable to the distinct properties of these mutations. The *C9ORF72* HRE is associated with incomplete and age-dependent penetrance and has been reported in both familial and sporadic ALS and FTD. Although the disease course in C9ORF72-related ALS is often of moderate duration, considerable variability exists [13]. The *TARDBP* p.A382T variant has been identified in both familial and sporadic cases and has been described as a pathogenic mutation; however, the clinical expression appears variable. While some reports have linked this mutation to a more rapid progression and reduced survival [14], *TARDBP* mutations are also known to exhibit reduced penetrance and, in many cases, a prolonged disease course. Therefore, the penetrance and phenotypic consequences of *TARDBP* mutations, particularly in individuals with additional pathogenic variants, remain insufficiently characterized. The differing characteristics of these two mutations may account, at least in part, for the observed variation in disease onset and progression among the individuals studied. Notably, the earlier age of onset and more aggressive disease course observed in some oligogenic cases suggest a potential synergistic effect. One particularly striking observation was that a monogenic *C9ORF72* carrier exhibited earlier symptom onset than their *C9ORF72/TARDBP*-positive parent, and a similar age of onset to extended relatives who also carried both mutations. These findings raise the possibility that oligogenic inheritance, in conjunction with other genetic, epigenetic, or environmental modifiers, may play a role in modulating disease expression and penetrance.

Pathologically, studies have suggested that the presence of the *C9ORF72* HRE modifies the burdens of TDP-43 pathology [3]. Indeed, several DPRs were found to promote TDP-43 aggregation *in vitro,* such as poly(GA) and poly(GR) [15–18]. Conversely, there is also evidence supporting a role for TDP-43 mislocalization in increasing DPR pathology [19]. When comparing individuals 1 and 2, the presence of an additional mutation in the *TARDBP* gene was associated with a more widespread distribution of pTDP-43 pathology, as well as poly(GR) inclusions. However, when comparing with additional *C9ORF72* carriers, the neuropathological burdens observed in the double mutation carrier (proband) were similar. In fact, the presence of a mutation in the *TARDBP* gene did not seem to significantly influence the severity of either pTDP-43 or DPR pathologies. In turn, it is evident that *C9ORF72* carriers can show pathological variability, as noted by high standard deviations for both pTDP-43 and DPR severity scores. Therefore, this could also be the case for *TARDBP* mutation carriers, with or without a coexisting mutation.

Previous reports have described ALS patients with combined *C9ORF72* and *TARDBP* mutations. In particular, the study by van Blitterswijk et al. [20] reported a family in which individuals with both mutations presented with an earlier age of onset compared to those carrying only the *TARDBP* variant. Although neuropathological data were not available, the clinical findings are consistent with the hypothesis of synergistic effects between mutations. These observations support the concept of oligogenic inheritance in a subset of ALS cases and are consistent with the patterns observed in our study. The inheritance pattern observed in this family further underscores the complexity of genetic transmission in ALS. DNA from the proband’s parents was unavailable, precluding determination of the precise inheritance mechanism. Nevertheless, the fact that four of five offspring carried both mutations, despite neither parent being affected, suggests incomplete or age-dependent penetrance. Both parents were beyond the typical age of onset, which may reflect non-manifesting carrier status. Incomplete penetrance has been well described for *C9ORF72* carriers [21] and has also been reported in familial cases of *TARDBP* mutations [6], though less frequently. The possibility that one or both parents carried pathogenic variants but remained asymptomatic due to absence of additional genetic or environmental factors cannot be excluded. These findings provide further support for a model of oligogenic inheritance in ALS, where interactions between pathogenic variants in different genes may influence disease onset, severity, and penetrance. Comprehensive genetic testing in familial ALS cases, along with careful phenotypic documentation, remains essential for understanding the mechanisms underlying variable disease expression and for informing genetic counseling.

To our knowledge, this is the first report to describe a very rare occurring *TARDBP and C9ORF72* double mutation with available postmortem data. Although a previous study observed a more aggressive disease course in double mutation carriers [22], this was not reflected in higher pathological burdens compared to *C9ORF72* carriers. This paves the way for further studies addressing the additive effects of *C9ORFf72* HRE and mutations in the *TARDBP* gene, as well as the interactions between both pathologies. Moreover, the availability of postmortem data in this unique case highlights the importance of thorough neuropathological assessments to confirm or refute synergistic pathological mechanisms. While TDP-43 proteinopathy is well recognized in both sporadic and familial forms of ALS, especially those involving *TARDBP* mutations, our results imply that a *C9ORF72* HRE might overshadow or mitigate the impact of a co-occurring *TARDBP* variant. At the same time, the possibility that *TARDBP* could exert subtle disease-modifying effects, undetectable in a single case, should not be discounted and warrants more extensive study. These insights pave the way for future investigations to systematically explore the additive or interactive effects of HRE *C9ORF72* and mutations in the *TARDBP* gene. Larger cohorts, ideally featuring detailed genetic, clinical, and postmortem data, are needed to clarify whether double heterozygosity leads to distinct disease trajectories, neuropathological patterns, or clinical presentations.

From a therapeutic standpoint, the co-occurrence of *C9ORF72* and *TARDBP* mutations presents a unique opportunity to unravel shared and potentially converging disease mechanisms. Both genes are central to RNA processing and cellular stress responses, including stress granule dynamics and nucleocytoplasmic transport. TDP-43, encoded by *TARDBP*, regulates a wide array of neuronal transcripts, and its dysfunction has been linked to broad disturbances in RNA homeostasis. Although direct regulatory interactions between *C9ORF72* and *TARDBP* have not yet been demonstrated, the disruption of common molecular pathways suggests that one may influence the pathogenicity of the other. Epigenetic mechanisms may also play a role. Methylation at the *C9ORF72* locus is known to modulate disease expression and could be affected by additional genetic variants such as *TARDBP* mutations. Whether such interactions amplify, mitigate, or reshape disease trajectories remains an open but compelling question. Understanding how these two mutations interact whether through additive, synergistic, or compensatory effects, could open new avenues for therapeutic intervention. Targeting shared downstream pathways, such as RNA metabolism, protein homeostasis, or stress response mechanisms, may offer a more effective strategy in cases with dual mutations. Furthermore, stratifying patients based on oligogenic profiles could guide the development of precision medicine approaches tailored to individual genetic backgrounds.

## Materials and methods

### Human cases

A total of 6 cases were used in the study: 5 C9orf72 expansion carriers including the Proband’s son, and 1 case with *TARDBP* and *C9ORF72* double mutation. Brain and spinal cord tissues from all cases were collected in the UZ Leuven brain biobank in accordance with the ethics review board upon written informed consent. Clinical assessment was performed by an expert neurologist. The diagnosis of ALS was based on the revised El Escorial criteria and the Awaji algorithm [23–25]. Brain and spinal cord autopsies were carried out by a board-certified neuropathologist. Microscopically, the diagnosis and staging of ALS was assessed by the regional presence phosphorylated TDP-43 (pTDP-43) pathology [1, 3]. The stages of pTDP-43 pathology were defined following previously defined criteria for ALS cases [3]. Alzheimer’s disease was excluded according to the NIA-AA criteria [26]. Primary age-related tauopathy, argyrophilic grain disease and age-related tau astrogliopathy were assessed according to previously published criteria [27–29].

### Immunohistochemistry

Histological examination was performed on 5-μm-thick sections cut from formalin-fixed, paraffin embedded tissue of the frontal cortex, cingulate gyrus, parietal cortex, occipital cortex, hippocampus, entorhinal cortex, hypothalamus, basal ganglia, amygdala, thalamus, midbrain, pons, medulla oblongata, cerebellum, spinal cord (cervical, thoracic, lumbar and sacral) and pre/postcentral gyrus. Poly(GA) and pTDP-43 immunostainings were partially performed automatically by means of the BOND-MAX automated IHC/ISH Stainer (Leica Biosystems, Wetzlar, Germany) using the Bond Polymer Refine Detection kit (DS9800, Leica Biosystems). Poly(GR) stainings were performed fully manually. For poly(GA), poly(GR) and pTDP-43, heat pretreatment (K800521-2, EnVision FLEX Target Retrieval Solution, Low pH; Agilent, Santa Clara, CA, USA) was performed by means of the PT Link system (Agilent). Subsequently, deparaffinization by going through a series of xylene and ethanol for rehydration was performed. To detect poly(GA) and poly(GR), an extra pretreatment with formic acid was performed to enhance the signal. For poly(GA) and pTDP-43, the next steps were performed using the Bond Polymer Refine Detection kit (DS9800) in the BOND-MAX automated IHC/ISH Stainer. For poly(GR), endogenous peroxidase activity was blocked by Dako REAL Peroxidase-Blocking Solution (S202386-2, Agilent). Primary antibody incubation was followed by a biotinylated secondary antibody and an avidin-biotinylated peroxidase complex (PK-6104, Vector Laboraties, Burlingame, CA, USA) for poly(GR) and visualized with diaminobenzidine (K346889-2, Agilent). Afterwards, hematoxylin counterstaining was performed. Primary antibodies used in this study were mouse monoclonal anti-poly(GA) clone 5E9 (MABN889, Merck Millipore, Billerica, USA) at a dilution of 1/1000 for 30 minutes, rat monoclonal anti-poly(GR) clone 5A2 (MABN778, Merck Millipore) at a dilution of 1/400 overnight, and rabbit polyclonal anti-pTDP-43 (pS409/410) (22309-1-AP, Proteintech, Illinois, USA) at a dilution of 1/5000 for 30 minutes. For neuropathological diagnosis, sections from the hippocampus, entorhinal and occipital cortex were additionally stained with anti-Aβ_17-24_ (4G8, Covance, Dedham, MA, USA) at a dilution of 1/5000 for 30 minutes and anti-phosphorylated tau protein (AT8, Thermo Scientific) at a dilution of 1/1000 for 30 minutes.

### Assessment of DPR and TDP-43 pathology

The assessment of both pathologies was performed as done previously [12]. The DPR and TDP-43 pathology in both cases was evaluated with a Leica DM2000 LED microscope (Leica Biosystems). A semiquantitative grading system was used by counting the total amount of pathology (including neuronal cytoplasmic and intranuclear inclusions, dystrophic neurites, diffuse granular cytoplasmic staining and pan-nuclear DPR expression) in a 200x microscopic field considered as the ‘hotspot area’. DPR and TDP-43 pathology was rated as ‘0’ if no pathology was present, as ‘1’ if 1 to 3 pathological lesions were present in the hotspot area, as ‘2’ if 4 to 7 pathological lesions were present in the hotspot area, as ‘3’ if 8 to 20 pathological lesions were present in the hotspot area, as ‘4’ if 21 to 50 pathological lesions were present in the hotspot area and as ‘5’ if more than 50 pathological lesions were present. Images were obtained by the Leica DM2000 LED microscope (Leica Biosystems) coupled to a Leica DFC 7000 T camera (Microsystems). Images were processed in ImageJ software (NIH, USA).

## Data Availability

All data relevant to the study are included in the article.
